# Suspected Group A Streptococcal Urethritis Following Orogenital Exposure in a Young Adult Male

**DOI:** 10.7759/cureus.115348

**Published:** 2026-08-28

**Authors:** Calvin Zhou, James Tran, Whinkie Leung, Gerard Laconsay, Mohamed N Jabri

**Affiliations:** 1 Medicine, Rocky Vista University College of Osteopathic Medicine, Ivins, USA; 2 Pediatrics, Alexian Brothers Hospital, Elk Grove Village, USA

**Keywords:** case report, dysuria, group a streptococcus, orogenital contact, sexual health, streptococcus pyogenes, urethritis

## Abstract

*Streptococcus pyogenes*, also known as group A Streptococcus (GAS), commonly causes pharyngitis and skin infection but rarely causes male genitourinary disease. We describe an uncircumcised man in his early twenties who developed progressive dysuria one day after receiving oral sex from his long-term, mutually monogamous partner, who reportedly had symptomatic GAS pharyngitis and was taking antibiotics. Examination documented urethral meatal erythema and tenderness without discharge. Leukocyte esterase was negative; urine microscopy showed 0-5 white blood cells and 5-10 red blood cells per high-power field, and urine culture yielded 20,000 colony-forming units per milliliter (CFU/mL) of beta-hemolytic GAS. Urine nucleic acid amplification tests (NAATs) for *Neisseria gonorrhoeae* and *Chlamydia trachomatis* were negative; several other causes of nongonococcal urethritis were not tested. Available records documented a 10-day course of oral amoxicillin, although the dose and timing relative to the culture result were unavailable. Symptoms improved within 48 hours and resolved within several days. The close timing between orogenital exposure and symptom onset, focal meatal findings, and quantitative GAS growth support the possible clinical relevance of the GAS isolate. However, the absence of a urethral specimen and incomplete pathogen testing prevent definitive classification as urethritis; the absence of paired isolates also prevents confirmation of direct transmission. This case emphasizes site-specific sexual history and targeted bacterial culture when initial testing is unrevealing.

## Introduction

*Streptococcus pyogenes*, also known as group A Streptococcus (GAS), is a human-adapted, gram-positive, beta-hemolytic coccus that causes a broad spectrum of diseases. Common manifestations include pharyngitis and superficial skin infection, whereas invasive disease and immune-mediated sequelae are less frequent but clinically significant complications [1]. Genitourinary infections caused by GAS are rare, particularly among adult men, and remain poorly characterized due to the limited number of reported cases.

Urethritis is most often evaluated in the context of established sexually transmitted pathogens, including *Neisseria gonorrhoeae*, *Chlamydia trachomatis*, and *Mycoplasma genitalium*. Nevertheless, a substantial proportion of nongonococcal urethritis cases remain idiopathic, and oral-genital contact can introduce organisms that typically colonize the respiratory tract [2,3]. Published reports have identified *S. pyogenes* as a rare cause of urethritis, balanitis, and balanoposthitis, with several authors proposing penile-oral transmission in selected cases [4-7]. Awareness of GAS as an uncommon potential genitourinary pathogen may be clinically useful because it can be missed when the exposure history is incomplete or bacterial culture is not obtained.

Current Centers for Disease Control and Prevention (CDC) guidance recommends objective evidence of urethral inflammation. When point-of-care microscopy is unavailable, a positive leukocyte esterase test on first-void urine or at least 10 white blood cells (WBCs) per high-power field (HPF) in spun first-void sediment supports urethritis; nucleic acid amplification tests (NAATs) for *Neisseria gonorrhoeae* and *Chlamydia trachomatis* should also be obtained [8]. These NAATs do not detect GAS. CDC reserves testing for less-common organisms for clinically suggestive or nonresponsive cases [8]. Site-matched bacterial sampling for suspected atypical infections is a case-informed extension rather than GAS-specific CDC guidance [4-7].

Against this background, we report a young adult man who developed progressive dysuria following orogenital exposure to his long-term partner, who reportedly had recently been diagnosed with GAS pharyngitis.

This case was previously presented as a poster and published as a meeting abstract at the Fall 2024 Rocky Vista University Research Symposium in November 2024.

## Case presentation

A man in his early twenties with no significant past medical history presented to an outpatient clinic with worsening dysuria and suprapubic discomfort. He reported symptom onset after receiving oral sex from his long-term partner, with whom he was in a mutually monogamous relationship. Mild pain with urination began one day after the encounter. Over the following two days, the pain became sharp, radiated along the urethra, and localized predominantly to the urethral meatus. By three days after exposure, pain peaked at approximately 7/10 during urination and prompted evaluation. Before receiving antibiotics, he had not used over-the-counter analgesics or other symptomatic treatments. Notably, he was uncircumcised and reported twelve prior sexual partners before his current mutually monogamous relationship, denying any additional sexual exposures during the relationship.

At the time of orogenital contact, his long-term partner had symptoms of streptococcal pharyngitis, reportedly had been diagnosed with GAS pharyngitis, and was taking antibiotics. The partner's diagnosis was based solely on the patient's report; the confirmation method, antibiotic agent, and duration of treatment before the encounter were unavailable. The partner was not independently evaluated or cultured as part of this case report, and no paired isolates were available for comparison. The patient denied sore throat, fever, rash, and urethral discharge. He reported no visible genital lesions other than redness centered on the urethral opening and penile tip. Examination documented erythema and tenderness of the urethral meatus without visible discharge. Vital signs and findings from a complete genitourinary examination were not available in the records reviewed.

Dipstick urinalysis showed clear yellow urine with a specific gravity of 1.005 or less and a pH of 6.5. Leukocyte esterase, nitrite, protein, glucose, bilirubin, and ketones were negative; trace lysed blood was present. Urine microscopy showed 0-5 white blood cells (WBCs) per high-power field (HPF), 5-10 red blood cells (RBCs) per HPF, and rare epithelial cells. Urine culture yielded beta-hemolytic GAS at 20,000 colony-forming units per milliliter (CFU/mL). The laboratory did not perform further routine susceptibility testing and noted penicillin as the drug of choice. The culture reports available for review did not document the collection method or organism-identification method.

Urine and throat NAAT results for *Neisseria gonorrhoeae* and *Chlamydia trachomatis* were negative. The urine result addressed urethral gonorrhea and chlamydia; the throat result documented pharyngeal screening but did not evaluate the cause of the patient's urethral symptoms. Screening for human immunodeficiency virus types 1 and 2 (HIV-1/2), hepatitis C virus (HCV) antibody, and syphilis by rapid plasma reagin (RPR) was nonreactive. No results were available for *Mycoplasma genitalium*, *Trichomonas vaginalis*, herpes simplex virus (HSV), or adenovirus. A urethral smear for Gram staining was not obtained; therefore, objective urethral inflammation and the presence or absence of intracellular Gram-negative diplococci could not be assessed microscopically. Vital signs and laboratory findings are summarized in Table 1.

**Table 1:** Vital signs and laboratory evaluation at presentation. CFU/mL, colony-forming units per milliliter; GAS, group A Streptococcus; HCV, hepatitis C virus; HIV-1/2, human immunodeficiency virus types 1 and 2; HPF, high-power field; NAAT, nucleic acid amplification test; RBC, red blood cell; RPR, rapid plasma reagin; WBC, white blood cell. Reference values for urine specific gravity, pH, and microscopy are typical adult intervals and may vary by laboratory because laboratory-specific ranges were unavailable in the records reviewed.

Examination	Patient result	Units	Reference or expected value
Vital signs	Not available in records reviewed	Not documented	Not applicable
Urine appearance	Clear yellow	Qualitative	Clear, yellow
Urine specific gravity	≤1.005	Unitless	1.005-1.030
Urine pH	6.5	Unitless	5.0-8.0
Leukocyte esterase	Negative	Qualitative	Negative
Nitrite	Negative	Qualitative	Negative
Protein, glucose, bilirubin, and ketones	Negative	Qualitative	Negative
Blood	Trace, lysed	Qualitative	Negative
White blood cells	0-5	Cells/HPF	0-5 cells/HPF
Red blood cells	5-10	Cells/HPF	0-2 cells/HPF
Epithelial cells	Rare	Qualitative	None to rare
Urine culture	Beta-hemolytic GAS, 20,000	CFU/mL	No growth
Susceptibility testing	Not performed; report noted penicillin as the drug of choice	Not applicable	Not applicable
Urine *C. trachomatis*/*N. gonorrhoeae* NAAT	Negative for both organisms	Qualitative	Negative
Throat *C. trachomatis*/*N. gonorrhoeae* NAAT	Negative for both organisms	Qualitative	Negative
HIV-1/2 screen	Nonreactive	Qualitative	Nonreactive
Rapid plasma reagin	Nonreactive	Qualitative	Nonreactive
Hepatitis C virus antibody	Nonreactive	Qualitative	Nonreactive

The available case record documented a 10-day course of oral amoxicillin. The exact dose and administration frequency were unavailable, and the patient could not independently verify the prescription details during later history confirmation. The records did not establish whether amoxicillin was started before or after the culture result or whether any additional empiric antimicrobial therapy was administered. Symptoms improved markedly within 48 hours and resolved completely within several days. Although symptoms disappeared early, he completed all 10 days of treatment. He did not return for an in-person follow-up visit, and no test-of-cure culture was obtained. The clinical course is summarized in Table 2.

**Table 2:** Relative clinical timeline from orogenital exposure through symptom resolution. CFU/mL, colony-forming units per milliliter; GAS, group A Streptococcus; HCV, hepatitis C virus; HIV, human immunodeficiency virus; HPF, high-power field; NAAT, nucleic acid amplification test; RBC, red blood cell; RPR, rapid plasma reagin; WBC, white blood cell.

Relative time	Clinical event
Exposure	Received oral sex from his long-term, mutually monogamous partner. The partner had pharyngitis symptoms, a patient-reported GAS diagnosis, and was already taking antibiotics.
One day after exposure	Mild pain with urination began.
Three days after exposure	The pain had become sharp and worsened with urination. It radiated along the urethra, was most intense at the external urethral meatus, and peaked at approximately 7/10. The patient contacted a physician.
Evaluation	The patient presented with dysuria and suprapubic discomfort. Examination documented localized erythema and tenderness at the urethral meatus without visible discharge. Vital signs and a complete genitourinary examination were unavailable. Urine and throat specimens and blood screening tests were obtained.
Laboratory results	Urinalysis showed trace lysed blood, 0-5 WBC/HPF, and 5-10 RBC/HPF. Urine and throat NAATs for *Neisseria gonorrhoeae* and *Chlamydia trachomatis* were negative. HIV, RPR, and HCV screening results were nonreactive. Urine culture yielded 20,000 CFU/mL of beta-hemolytic GAS.
Treatment	The available record documented a 10-day course of oral amoxicillin; the exact dose, frequency, timing relative to the culture result, and any additional empiric therapy were unavailable.
Approximately 48 hours after starting treatment	Symptoms improved markedly.
Within several days of treatment	Symptoms resolved completely. The patient completed the full 10-day antibiotic course despite early improvement. He did not return for formal follow-up, and no test-of-cure culture was obtained.

## Discussion

This case describes urethral symptoms and urinary GAS growth occurring shortly after reported orogenital exposure from a long-term partner who, according to the patient, had symptomatic GAS pharyngitis. GAS is not conventionally classified as a sexually transmitted infection (STI), although sexual contact may provide a route for mucosal inoculation. The short interval between exposure and symptom onset, focal meatal pain and inflammation, quantitative GAS growth in urine, negative urine gonorrhea/chlamydia NAAT, and improvement during amoxicillin therapy supported the possible clinical relevance of the isolate and were compatible with oropharyngeal-to-genital inoculation. However, treatment response is not pathogen-specific, and the unknown urine collection method, absence of a urethral specimen, incomplete testing for other causes, and lack of paired isolates prevent confirmation of urethritis, causation, or person-to-person transmission. The reported exclusive relationship made another recent sexual exposure less likely by history but does not provide microbiologic confirmation of transmission.

Prior reports document rare genital GAS infections occurring in temporal association with sexual exposure but do not establish GAS as a conventional STI. Edwards and Carne reviewed oral sex as a route for nonviral sexually transmitted infections and noted that respiratory organisms, including streptococci, may be transmitted orogenitally [4]. Minami et al. characterized four genetically distinct GAS isolates from balanoposthitis cases with suspected acquisition following penile-oral contact [5]. Norimatsu and Ohno reported GAS balanoposthitis in a 31-year-old man and emphasized its inclusion in the differential diagnosis [6]. More recently, Bartilotti Matos et al. described *Streptococcus pyogenes* urethritis with GAS recovered from urine and urethral exudate; however, concurrent herpes simplex virus type 2 infection complicated causal attribution [7]. Female genital tract reports, including severe pelvic inflammatory disease and an invasive GAS case associated with an intrauterine device and oral sex, demonstrate that genital infection can occur in varied clinical contexts [9,10].

Differential diagnosis and microbiologic interpretation

The differential diagnosis of dysuria and urethral symptoms in a sexually active man is broad. Infectious causes include *N. gonorrhoeae*, *C. trachomatis*, *M. genitalium*, *T. vaginalis*, herpes simplex virus, and adenovirus; noninfectious causes include chemical irritation and mechanical trauma [2,8]. Orogenital exposure is relevant because respiratory organisms such as *Haemophilus influenzae*, *Streptococcus pneumoniae*, and other uncommon bacteria have been associated with urethritis [2,3]. Localized meatal erythema raised the possibility of overlapping balanitis or balanoposthitis, but diffuse inflammation of the glans or foreskin was not documented. Suprapubic discomfort and microscopic hematuria also left cystitis in the differential. Urine NAAT was negative for gonorrhea and chlamydia, but testing for several other recognized causes of nongonococcal urethritis was not documented. The negative throat NAAT documented pharyngeal screening but did not determine the cause of the urethral symptoms.

The patient had urethral symptoms and meatal erythema, but no documented discharge or positive first-void leukocyte esterase result, and urine microscopy reported 0-5 WBCs/HPF. When urethral microscopy is unavailable, CDC criteria use at least 10 WBCs/HPF in spun first-void urine sediment as objective evidence of urethritis [8]. Because the collection method and whether the sediment was spun were not documented, this criterion cannot be applied with full confidence. Rare epithelial cells and quantitative GAS growth support possible clinical relevance, but 20,000 CFU/mL from an unspecified urine specimen cannot by itself distinguish urethral infection, bladder bacteriuria, or contamination. The Infectious Diseases Society of America definition of asymptomatic bacteriuria applies to patients without urinary symptoms and therefore does not provide a diagnostic cutoff for this symptomatic presentation [11]. No urethral Gram stain, urethral culture, or repeat culture was obtained. The findings are therefore compatible with, but not definitive for, GAS urethritis.

Potential presentation and transmission in women 

Recognition of GAS infection in females is also an important consideration when evaluating suspected sexually associated genitourinary disease, as acquisition and transmission pertain to both biological sexes. In adult women, localized genital GAS infection has been reported as vulvovaginitis with vulvar or vaginal pain, burning, pruritus, dyspareunia, and watery, yellow, or purulent discharge [12]. Rare ascending or invasive infection may present with pelvic or abdominal pain, fever, gastrointestinal symptoms, endometritis, salpingitis, bacteremia, hypotension, or shock [9]. Reported contexts include sexual contact, household respiratory or skin GAS infection, vaginal atrophy, childbirth, intrauterine device use, and gynecologic procedures [9,10,12]. These associations do not establish GAS as a conventional STI as genital carriage appears to be uncommon. When common causes of vaginitis, cervicitis, or pelvic inflammatory disease are not identified and exposure raises concern for GAS, vaginal or cervical bacterial culture may be considered; and blood cultures are appropriate when systemic illness is present [9,12]. Paired partner cultures and molecular typing would be required to confirm a specific transmission route.

Potential post-streptococcal complications

Acute rheumatic fever (ARF) and post-streptococcal glomerulonephritis (PSGN) are delayed immune-mediated sequelae established primarily after GAS pharyngitis or skin infection [1,13,14]. Their occurrence after genital GAS infection has not been established, and the cited urethritis and balanoposthitis reports did not describe either complication [5-7]. No genital GAS-specific guidance supports routine cardiac or renal surveillance after clinical resolution. Evaluation should instead be symptom-driven, including for dark urine, edema, reduced urine output, migratory joint symptoms, cardiorespiratory symptoms, rash, or involuntary movements. Although adequate treatment of pharyngeal or skin GAS helps prevent ARF [13], that evidence cannot be extrapolated to genital infection, and evidence remains insufficient to determine whether antibiotics prevent PSGN [14].

Potential relevance of uncircumcised status

Uncircumcised status was documented but should not be interpreted as a risk factor in this case. Circumcision can alter the genital microbiota [15], but the cited study did not evaluate GAS, and adult foreskin histology does not support a simple weaker-barrier explanation [16]. Without documented balanoposthitis or a subpreputial culture, this report cannot determine whether foreskin status influenced exposure, colonization, or susceptibility.

Potential pathogenic mechanisms

Known GAS virulence mechanisms offer biological plausibility but were not demonstrated in this isolate. M protein can inhibit complement-mediated opsonization, Protein F can promote fibronectin-dependent epithelial attachment, and streptolysin O can contribute to tissue injury [17-19]. Because the isolate was not genotyped or tested for virulence-factor expression, these mechanisms should not be interpreted as case-specific findings.

Treatment considerations

No evidence-based regimen has been established specifically for GAS urethritis because published cases are rare [5-7]. The 10-day amoxicillin course in this patient should therefore be reported as the treatment received, not as a validated urethritis regimen. After GAS identification, penicillin or amoxicillin is a conservative pathogen-directed choice by extrapolation from GAS pharyngitis guidance, which identifies these agents as preferred therapy and reports no clinical GAS isolates resistant to penicillin or cephalosporins [20]. For a patient with a true penicillin allergy, selection should account for reaction type and, when available, susceptibility data. Pharyngitis alternatives include cephalexin or cefadroxil for non-immediate reactions and clindamycin, clarithromycin, or azithromycin when beta-lactams are inappropriate; however, macrolide and clindamycin resistance varies geographically and temporally [20]. Urethritis-specific dose and duration data are unavailable. Persistent or recurrent symptoms should prompt reassessment for an alternative or additional diagnosis.

Limitations

The principal limitation is that neither the diagnosis of urethritis nor the route of transmission can be confirmed. The patient did not meet the commonly used urinalysis or discharge criteria for objective urethral inflammation, and no urethral smear for Gram staining or urethral culture was obtained. The urine collection and organism-identification methods were not documented, so the quantitative culture cannot exclude a false-positive result from periurethral or genital contamination or fully distinguish urethral infection from cystitis. The partner was not independently tested or cultured; her diagnostic method, isolate, antibiotic, treatment duration before exposure, and strain type were unavailable, and no paired isolates were available for comparison. Vital signs and a complete genitourinary examination were not available. Detailed chronology, pain severity, circumcision status, relationship exclusivity, and partner symptoms were clarified retrospectively and are subject to recall bias. Testing for *Mycoplasma genitalium*, *Trichomonas vaginalis*, herpes simplex virus, and adenovirus was not documented. The timing of amoxicillin relative to culture results, any additional empiric therapy, exact dose and frequency, long-term follow-up, and test-of-cure culture were unavailable. These limitations preclude conclusions about direct transmission, a causal role for the foreskin, optimal treatment, or delayed poststreptococcal risk.

Sexual health and primary care implications

The sexual health significance of this case extends beyond whether GAS should be labeled as an STI. A more clinically useful approach is to recognize GAS as a potentially sexually transmissible organism in selected exposure settings. A focused and nonjudgmental history should document the type of sexual contact, anatomic sites exposed, interval between exposure and symptoms, barrier use, partner symptoms or confirmed infections, recent antibiotic use, and the possibility of additional partners. This information can guide site-specific testing and help distinguish urethritis from cystitis, balanitis, mechanical irritation, or other genital disease.

CDC guidance uses a positive leukocyte esterase result in first-void urine or at least 10 WBCs/HPF in spun first-void sediment as objective evidence of urethral inflammation when microscopy is unavailable [8]. Because specimen type affects interpretation, the collection method should be documented. NAATs for gonorrhea and chlamydia are recommended even when symptoms occur without objective inflammation, but a negative result excludes only those tested organisms [8]. For persistent or recurrent symptoms after initial empiric treatment, CDC recommends testing for *Mycoplasma genitalium* and conditionally recommends testing for *Trichomonas vaginalis*; testing for less common organisms is reserved for suggestive clinical features or failure of recommended therapy [8]. When a urinary tract infection or an atypical bacterial process remains in the differential, urine culture or a site-matched bacterial culture may be considered an extension of routine clinical reasoning and published case experience, rather than a GAS-specific CDC algorithm [4-7]. Table 3 summarizes the role and limitations of commonly selected tests.

**Table 3:** Selected tests used in urethritis and sexual-health evaluation and their diagnostic gaps. *C. trachomatis*, *Chlamydia trachomatis*; GAS, group A Streptococcus; HCV, hepatitis C virus; HIV, human immunodeficiency virus; HSV, herpes simplex virus; *M. genitalium*, *Mycoplasma genitalium*; *N. gonorrhoeae*, *Neisseria gonorrhoeae*; NAAT, nucleic acid amplification test; *T. vaginalis*, *Trichomonas vaginalis*.

Test	Detects or evaluates	Important limitations	Reference(s)
First-void urinalysis	Positive leukocyte esterase or at least 10 WBCs/HPF in spun sediment can document objective urethral inflammation when urethral microscopy is unavailable.	Does not identify a pathogen; a negative result does not exclude gonorrhea or chlamydia in a symptomatic man.	[8]
Site-specific *N. gonorrhoeae*/*C. trachomatis* NAAT	Gonorrhea and chlamydia at the sampled anatomic site; urine is the preferred specimen for males.	Does not detect GAS or other causes, and the result applies only to the sampled site.	[8]
Additional pathogen-directed testing	*M. genitalium*, *T. vaginalis*, HSV, adenovirus, or other less-common causes selected according to persistence, recurrence, exposure, and clinical findings.	Not included in every initial evaluation; indications, specimen type, and test availability vary.	[8]
Targeted bacterial culture	Urinary or genital bacteria, including GAS when present; specimen should match the suspected site.	Interpretation depends on collection method, organism burden, competing diagnoses, and contamination risk.	[4-7]
Serologic screening	HIV and syphilis testing for men with nongonococcal urethritis; HCV screening at least once for most adults.	Supports broader sexual-health care but generally does not identify the cause of acute urethral symptoms.	[8]

No evidence-based partner-management guideline exists for GAS urethritis. General urethritis guidance recommends avoiding sexual intercourse until treatment is completed and symptoms have resolved [8]. Applied cautiously, temporary abstinence or barrier use until both partners have completed indicated treatment and are asymptomatic is reasonable, but this is extrapolated rather than GAS-specific. Partners should be evaluated according to their symptoms and the documented infection site; evidence does not support automatically applying expedited partner therapy protocols for gonorrhea or chlamydia to GAS. The most direct evidence in reported male genital GAS cases involves penile-oral exposure [5-7], and current data are insufficient to estimate transmission risk through vaginal or anal intercourse. 

Beyond partner management, Table 4 integrates CDC urethritis guidance with case-informed steps for considering GAS and other atypical organisms [4-8].

**Table 4:** Practical algorithm for evaluating symptomatic male urethritis, including nontraditional causes. *C. trachomatis*, *Chlamydia trachomatis*; GAS, group A Streptococcus; HSV, herpes simplex virus; *M. genitalium*, *Mycoplasma genitalium*; *N. gonorrhoeae*, *Neisseria gonorrhoeae*; NAAT, nucleic acid amplification test; *T. vaginalis*, *Trichomonas vaginalis*.

Step	Core action	When to broaden beyond common causes
1. History and examination	Assess dysuria, discharge, meatal or glans inflammation, lesions, urinary symptoms, systemic illness, and oral, vaginal, and anal exposures.	Oral exposure involving a partner with pharyngeal infection or focal meatal/glans findings should broaden the differential.
2. Initial testing	Document inflammation when possible; obtain urinalysis and urine *N. gonorrhoeae*/*C. trachomatis* NAAT, with additional exposed-site testing as indicated.	Add urine or site-specific bacterial culture when cystitis or an atypical bacterial process is plausible.
3. Reassess negative or persistent cases	Consider *M. genitalium*, *T. vaginalis*, HSV, adenovirus, cystitis, balanitis/balanoposthitis, chemical irritation, and trauma.	Revisit GAS or other oral bacteria when exposure history supports them and obtain an appropriate culture if not already collected.
4. Treat and follow	Use pathogen-directed therapy when possible and reassess persistent or recurrent symptoms.	Tailor partner evaluation and temporary sexual precautions to the identified organism, symptoms, and evidence base.

In primary care settings, this case highlights the value of a comprehensive, site-specific sexual history and selectively targeted testing. A negative gonorrhea/chlamydia NAAT may be reassuring for those pathogens but does not evaluate all causes of urethral symptoms after oral-genital exposure. When the clinical context supports an atypical bacterial process, culture may identify an organism not detected by molecular STI testing and support narrower antimicrobial therapy. Greater recognition and careful reporting of similar cases may help clarify whether sexually associated genital GAS infections are underdiagnosed and inform future testing and partner-management recommendations.

## Conclusions

Group A Streptococcus is a rare potential cause of male genitourinary symptoms. In this patient, the close timing between reported orogenital exposure and symptom onset, focal meatal findings, and quantitative GAS growth in urine supported the possible clinical relevance of the isolate. However, the absence of objective evidence of urethral inflammation, the lack of a urethral specimen, and the unknown urine collection method prevented a definitive classification as urethritis. A focused, site-specific sexual history should be paired with urinalysis and appropriate NAATs; bacterial culture may be useful when cystitis or an atypical bacterial infection remains under consideration.

Although these findings are compatible with oropharyngeal-to-genital inoculation, they do not establish that GAS caused urethritis or that direct sexual transmission occurred, nor do they justify classifying GAS as a conventional STI. The risk of delayed immune-mediated complications following genital infection remains unknown. Future reports should document specimen type and collection method, objective evidence of urethral inflammation, testing for established sexually transmitted and other nongonococcal causes, paired partner cultures with strain comparison, precise treatment timing and dosing, and clinical follow-up. Greater awareness in primary care may improve recognition, targeted testing, antimicrobial stewardship, and documentation of similarly unusual infections.
